# Assessment of hearing in a municipal public school student population

**DOI:** 10.1590/S1808-86942011000600007

**Published:** 2015-10-19

**Authors:** Janaina Cândida Rodrigues Nogueira, Maria da Conceição Mendonça

**Affiliations:** 1 Master's degree (physician); 2 Speech therapist, Nossa Senhora do Rosário Clinic

**Keywords:** audiometry, hearing, students

## Abstract

**Abstract:**

Children need good hearing for adequate learning. Hearing screening in students is important because it allows cases that go unnoticed by the family may be detected.

**Aim:**

To evaluate hearing in children aged 4-15 years - public school students from pre-first to the fifth grade in the city of Cabedelo, Paraíba state.

**Material and Methods:**

A prospective study of 98 students referred by teachers of 18 public schools in Cabedelo, from June 2007 to June 2010. Students were examined by an otolaryngologist and underwent audiometric testing.

**Results:**

Eighteen schools were enrolled in this study. The student's age ranged from 4 to 15 years; 62% were male and 38% were female. Otolaryngological tests were normal in 85% of cases, and abnormal in 15% of cases. The most frequent findings were ear wax and tympanic membrane retraction. Audiometry demonstrated normal hearing in 66% of children and impaired hearing in 34%.

**Conclusion:**

Hearing Screening in school children is an important method for diagnosing hearing loss, especially in low-income populations.

## INTRODUCTION

Adequate hearing is one of the main factors for good psychosocial development, by which individuals may express their thoughts, feelings, and wishes, and acquire life experience and knowledge. Therefore, hearing loss needs to be diagnosed early for prompt therapy. Public health policies have defended and developed the Neonatal Hearing Screening Program (PTANU); hearing screening is also recommended in school-aged children^1^.

Early in life infants need to listen in order to acquire language; often hearing loss is detected at school by teachers. Araujo^2^ argues that hearing evaluation in school-aged children is necessary to detect and correct hearing loss at an early age – this is a part of basic health measures for school-aged children.

Children require normal hearing – or adequately corrected hearing – to facilitate the formal education process. Hearing loss in infancy may be congenital or acquired; a likely cause should be investigated, although in most cases it remains unclear^3^.

Often an early diagnosis is not made because parents and family members fail to notice any hearing loss – specialists are then sought when the infants are aged over 2 years. Many children reach school age without a diagnosis of hearing loss; they are often considered hyperactive children, poor performers, or non-adapted to school, and are sent to special schools when in fact the problem is hearing loss^4^. The role of schools and teachers in these cases is extremely important.

There is, however, no systematic approach for screening in schools by teachers. Additional research by physicians, speech therapists, and teachers is needed, given the importance of this topic.

Thus, the purpose of this study was to assess the hearing of children aged from 4 to 15 years, from preschool-1 to 5^th^ grade in public education schools in the city of Cabedelo, Paraiba state (PB), to evaluate the importance of school hearing screening.

## MATERIALS AND METHODS

The sample comprised 98 students from preschool-1 to 5^th^ grade referred to us by teachers in 18 public schools in Cabedelo, PB, from June 2007 to June 2010. The child's parent or caretaker signed a free informed consent form. The institutional review board approved the study (no. 505/2010). Children were screened by teachers and then enrolled in the study regardless of the medical and phonological examination. Screening was done by observing teaching activities such as dictation, reading, participation in group activities such as music classes, and others. Children that did not perform adequately were included in the study, and underwent an otorhinolaryngological examination and pure tone audiometry, irrespective of age. If wax was found in the outer ear or middle ear disease was present, treatment was given before testing.

Audiometry was done in an acoustic booth (measuring 2 m width by 2 m length by 1.8 m height, with antireverberating foam protection) installed in an acoustically treated room. A Damplex DA64 Interacoustic audiometer, calibrated in January 2010 according to the Regulation 19/98 of the Ministry of Health, was used. Auditory thresholds were measured at 1, 2, 3, 4, 6, and 8 kHz by air and bone conduction. The statistical analysis and the charts were done using the Microsoft Office Excel/2003 software.

## RESULTS

**Graph 1:**
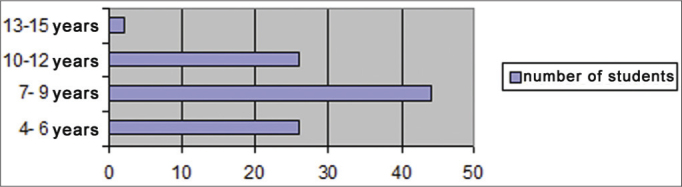
Number of students per age group.

**Graph 2:**
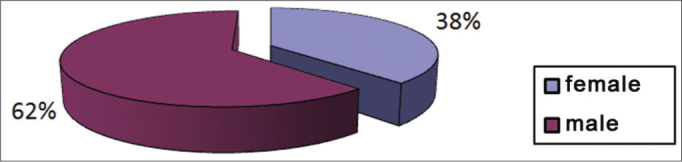
Distribution of students according to sex.

**Graph 3:**
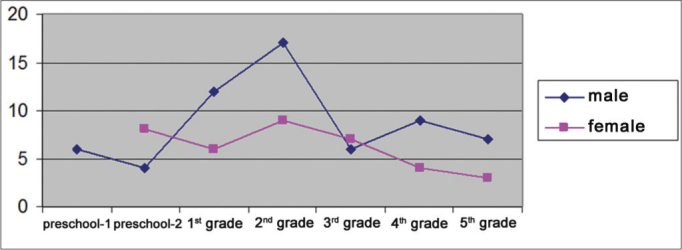
Distribution of students according to sex and school grade.

There were 98 students aged from 4 to 15 years (Graph 1), of which 37 were female (38%) and 61 were male (62%) (Graph 2), who underwent an otorhinolaryngological examination and audiometry. Graph 3 presents the age and sex distribution of subjects.

There were 18 public schools involved in this study. Graph 3 shows the number of male and female students according to school grades.

**Graph 4:**
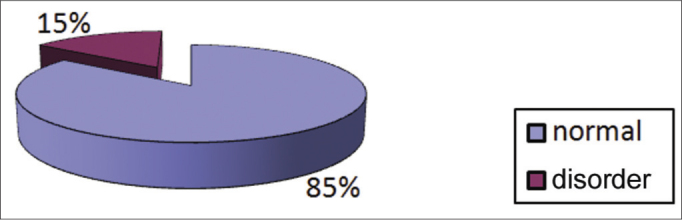
Distribution of students according to the findings of the otorhinolaryngological examination.

**Graph 5:**
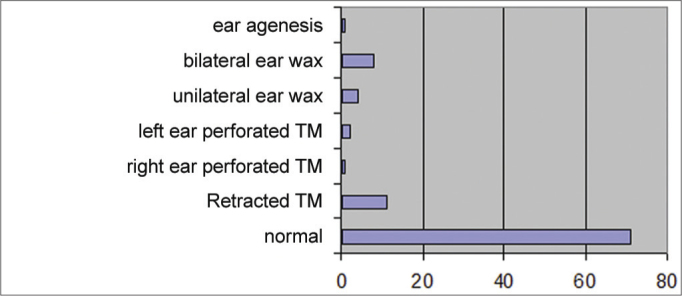
Findings of the otorhinolaryngological examination.

Examination and testing was carried out from June 2007 to June 2010. The otorhinolaryngologic examination was normal in 85% of cases and abnormal in 15% of cases (Graph 4); the main causes of this were excess wax in retraction of the tympanic membrane (Graph 5).

**Graph 6:**
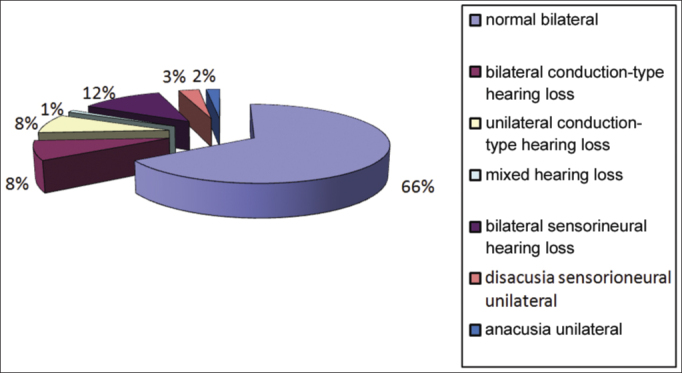
Audiometry findings.

Bilateral normal hearing was observed in 66% of subjects; bilateral sensorineural hearing loss was seen in 12% of subjects, bilateral conductive hearing loss was present in 8% of subjects, unilateral conductive hearing loss was found in 8% of subjects, unilateral sensorineural hearing loss was seen in 3% of subjects, mixed unilateral was found in 1% of subjects, and unilateral anacusis was observed in 2% of subjects (Graph 6). Bilateral hearing loss was more frequent than unilateral hearing loss.

**Graph 7:**
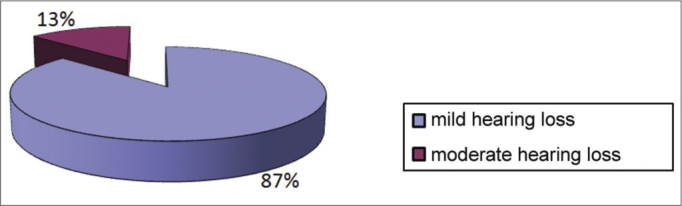
Distribution of students according to the degree of conductive hearing loss.

**Graph 8:**
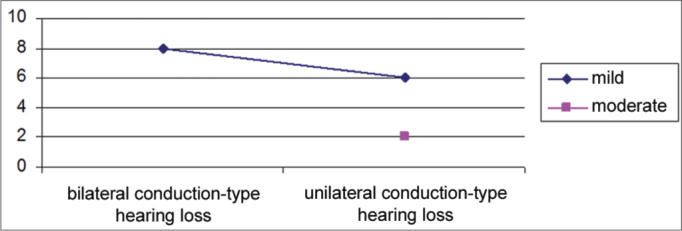
Distribution according to the grade of conductive hearing loss and ear affected.

Conductive hypoacusis was mild in 87% of cases and moderate in 13% of cases (Graph 7). It was bilateral in 8 cases, all of which were mild grade. It was monaural and of mild grade in 6 subjects, and moderate grade in 2 (Graph 8).

**Graph 9:**
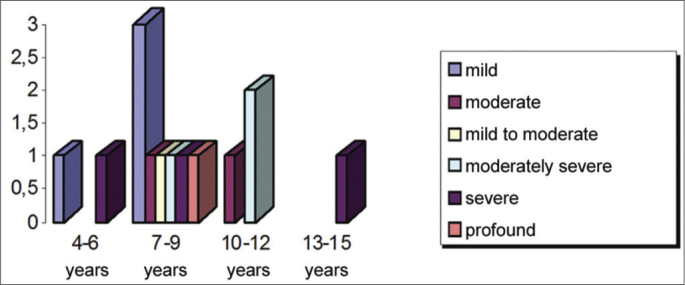
Distribution of students according to the type of sensorineural hearing loss and age group.

Sensorineural hearing loss was bilateral in 12 subjects and unilateral left in 3 subjects. It ranged from mild to profound; Graph 9 shows the distribution of hearing loss by age group.

## DISCUSSION

There is currently growing interest in the early prevention of diseases, which has led to many previously considered untreatable conditions to be better approached, benefitting individuals and improving their quality of life.

These policies have significantly supported the early detection and treatment of hearing loss, resulting in improvements for individuals and fewer problems in psychosocial development. Tucker^5^ has argued that hearing is an ability that may be apprehended; thus, hearing screening in schools – especially in children with unilateral mild to moderate hearing loss – should be considered.

Rabinovich^6^ has stated that early identification of hearing loss and adequate corrective measures foster speech, language, social, psychic, and educational development, and a more satisfactory outcome.

There were 98 students aged from 4 to 15 years, of which 37 were female (38%) and 61 were male (62%), who underwent an otorhinolaryngological examination and audiometry.

Most of the referred students were aged 4 to 9 years, which is the time when children learn to read and write; thus, adequate hearing is important for linguistic development and participation in school activities.

There were more male students, probably because they face more difficulties at school during this initial phase of learning. Although few studies have shown any interference of gender on phonological awareness, the notion that words consist of a sequence of sounds, researchers have found that girls tend to produce more when expressing themselves orally, to have better auditory discrimination and visual/motor coordination, and to perform better in initial acquisition of reading and writing^7^. Souza^8^ studied school children in the 1^st^ year of 1 grade in the city of Natal and found that girls were more successful in all phonemic segmentation and synthesis tasks and performed better in phonemic awareness abilities, although this difference between sexes was not statistically significant. Capellini et al.^9^ found that there were more male children with dyslexia compared to female children.

The otorhinolaryngological examination was normal in 85% of the sample and abnormal in 15%. The predominant findings were excess was and a retracted tympanic membrane, suggesting tube dysfunction – common in this age group.

The otoscopic findings were excess ear wax and retraction of the tympanic membrane, suggesting tube dysfunction. Vieira et al.^1^ has reported that preschool and school children usually present hearing loss because of excess wax, foreign bodies, otitis externa, and otitis media with effusion.

Rodrigues's^10^ study in public and private schools showed that 52 children were normal hearing and 25 had impaired hearing. Araújo et al.^2^ (2002) analyzed 121 school-aged children in Goiania and found that most audiometries were normal (76%), and 24% were abnormal; our results are similar, with most students having normal hearing.

In that same study, mild conductive hearing loss was found in half of the cases, and mild sensorineural hearing loss was present in 7% of students. In our sample, conductive hearing loss did not predominate, but was somewhat more frequent than sensorineural hearing loss. Conductive and sensorineural bilateral hearing loss was present in 20 students; unilateral hearing loss was found in 14 students – mild and moderate grade hearing loss predominated.

Mild to moderate hearing loss may often go unnoticed by teachers, parents, and even students themselves; this is more important when hearing loss is unilateral. Therefore, concern by teachers and school hearing screening by speech therapists become essential; even unilateral mild to moderate hearing loss may result in school underperformance and have significant consequences for the child's life to the extent that psychopedagogic monitoring may be needed^4^.

Sensorineural hearing loss was more frequent in the 7 to 9 year age group. In these subjects, hearing loss was detected in school screening. In public schools, this is the age at which students learn how to read and write, and good hearing is essential. Three students had severe bilateral hearing loss; one of these was in the 13 to 15 year age group, which underlines the importance of hearing diagnosis. Family members, particularly in less favored social classes, may not be attuned to perceiving hearing loss. Cano & Silva^11^ found that the parents of 10 children with hearing loss did not identify this condition; they looked at the education level of the parents and found that most had at most some high school education, and that the family income was between 6 and 10 minimum salaries in only one family. Thus, neonatal hearing screening, or if absent school hearing screening, may detect auditory disorders in the pediatric population, and open the opportunity for better studying and quality of life for these people.

## CONCLUSION

A normal otorhinolaryngological examination and audiometry predominated in the study sample. There was more screening for male students aged 4 to 9 years. Conductive hearing loss cases were mostly mild, whereas mild cases did not predominate among subjects with sensorineural hearing loss, which was found mainly in school children aged from 7 to 9 years. Thus, we conclude that school hearing screening is an important approach for the diagnosis of hearing loss, especially in low-income populations.
